# Temporal dynamics of IgG-mediated immunometabolic dysfunction: from acute obesity to chronic aging

**DOI:** 10.3389/fimmu.2025.1661391

**Published:** 2025-12-04

**Authors:** Sohyun Kim, Junghyun Kim, Hyung-Lae Lee, Man S. Kim

**Affiliations:** 1 Translational-Transdisciplinary Research Center, Clinical Research Institute, Kyung Hee University Hospital at Gangdong, College of Medicine, Kyung Hee University, Seoul, Republic of Korea; 2 Department of Medicine, Kyung Hee University College of Medicine, Seoul, Republic of Korea; 3 Division of Tourism & Wellness, Hankuk University of Foreign Studies, Yongin-si, Gyeonggi-do, Republic of Korea; 4 Department of Urology, School of Medicine, Kyung Hee University, Seoul, Republic of Korea

**Keywords:** IgG, FcRn, metabolic dysfunction, insulin resistance, adipose tissue inflammation, immunometabolism

## Abstract

Immunoglobulin G (IgG) is traditionally recognized as a circulating immune effector; however, recent discoveries have revealed that IgG accumulates in adipose tissue—up to 16-fold above plasma levels—and functions as a critical mediator of metabolic dysfunction in obesity and aging. This review summarizes evidence showing that adipocyte IgG accumulation occurs via neonatal Fc receptor (FcRn)-mediated uptake and directly competes with insulin for receptor binding through Fc-CH3 domain interactions. IgG initiates tissue-specific inflammatory responses. Functional outcomes depend on glycosylation patterns: sialylated IgG (e.g., control IgG) signals anti-inflammatory pathways via DC-SIGN and CD22, whereas hyposialylated IgG (e.g., disease-associated IgG) activates endothelial FcγRIIB receptors, impairs insulin transcytosis, and promotes vascular insulin resistance. This mechanism may help explain the limited success of conventional anti-inflammatory treatments for metabolic diseases. The timeline of IgG-mediated effects progresses through acute inflammation (weeks), subacute deposition and insulin interference (months), and chronic fibrosis (years). Notably, FcRn antagonists can reverse insulin resistance, while restoration of IgG sialylation using sialic acid precursors improves function without depleting antibodies. These findings suggest that IgG dysfunction occurs at the intersection of obesity, aging, and metabolic disease, offering new biomarkers and therapeutic targets. Glycosylation profiling enables the discrimination between insulin-sensitive and -resistant individuals with similar body mass indices, supporting precision medicine approaches. This paradigm shift, from cell-centric to antibody-mediated models, reframes our understanding of metabolic disease pathogenesis and offers novel treatment strategies.

## 1 Introduction: the immunometabolic revolution

Once separate disciplines, immunology and metabolomics are now converging to revolutionize our understanding of chronic diseases. While chronic low-grade inflammation has long been recognized as a feature of obesity-related metabolic dysregulation (1, 2), the exact molecular mechanisms by which immune system activation drives metabolic diseases remain unclear. Traditional paradigms have highlighted immune cell infiltration and cytokine release as key instigators of metabolic inflammation (3, 4). The seminal work of Xu et al. and Weisberg et al. in 2003 first established that macrophage infiltration of adipose tissue is a key pathological process in obesity, as obese adipose tissue is characterized by 40–60% macrophage infiltration compared to only 10–15% in lean adipose tissue (3, 4).

However, recent studies have fundamentally challenged this cell-centered view by demonstrating that immunoglobulin G (IgG), the most prevalent circulating antibody, plays a direct and previously unknown role in metabolic regulation. This paradigm has been triggered by three main breakthroughs: first, the demonstration that IgG selectively accumulates in metabolically active tissues through receptor-mediated mechanisms (5); second, the discovery of direct molecular interactions between IgG and metabolic signaling pathways (6); and third, the recognition that IgG-mediated mechanisms have both protective and pathogenic roles in metabolic diseases (7).

Reframing IgG as a metabolic regulator involves a shift from viewing antibodies solely as defense molecules to recognizing them as active contributors to tissue homeostasis and disease. Compared to the traditional focus on cellular immune infiltrates, this new understanding positions IgG as a soluble immune mediator at the center of metabolic dysfunction. The consequences extend far beyond intellectual interest, as they provide a mechanistic understanding of the rising global burden of metabolic diseases and yield novel therapeutic targets that promise to dissociate beneficial immune functions from adverse metabolic consequences.

While individual aspects of antibody biology—obesity-driven B cell dysfunction, autoantibody generation during aging, and immunosenescence—have been reviewed previously, the synthesis connecting these processes is lacking. Most studies have treated obesity- and age-driven metabolic dysregulation as separate processes, while there are recent studies, which identify similarities of obesity and ageing mechanisms (8, 9). However, the evidence suggests overlapping mechanisms. Additionally, antibody-mediated pathology has been under-studied relative to cellular immunity, particularly with respect to kinetics and therapeutic targeting.

This review aims to fill these gaps by proposing a shared temporal model of IgG-mediated immunometabolic dysfunction that encompasses both acute obesity-driven changes and chronic age-related decline. While direct temporal studies comparing obesity and aging are limited, we synthesize parallel evidence from both conditions to construct this integrative framework. Rather than scrutinizing separate mechanisms, we integrated the molecular, tissue-specific, and systemic levels of IgG dysfunction. We further highlighted the translational relevance of this model by illustrating how temporal knowledge of IgG pathology can inform precision medicine and multi-target therapies.

Our findings suggest that IgG dysfunction progresses along a predictable temporal trajectory linking obesity and aging, with the potential to treat various age-related diseases using antibody-targeted therapy. This perspective also accounts for the failure of traditional anti-inflammatory therapies at advanced stages of disease and identifies key windows of opportunity for effective treatment.

Explaining how IgG transitions from a protective immune factor to a metabolic disruptor can inform us about why some but not all individuals develop insulin resistance with similar environmental exposures, and why aging is inescapably associated with metabolic decline (10, 11). Additionally, the identification of specific IgG receptor pathways involved in metabolic regulation offers the potential for precise interventions using available pharmaceutical approaches originally developed for autoimmune diseases.

## 2 FcRn–IgG axis in metabolic disease

### 2.1 Obesity: acute IgG accumulation and insulin interference

**Figure 1:**
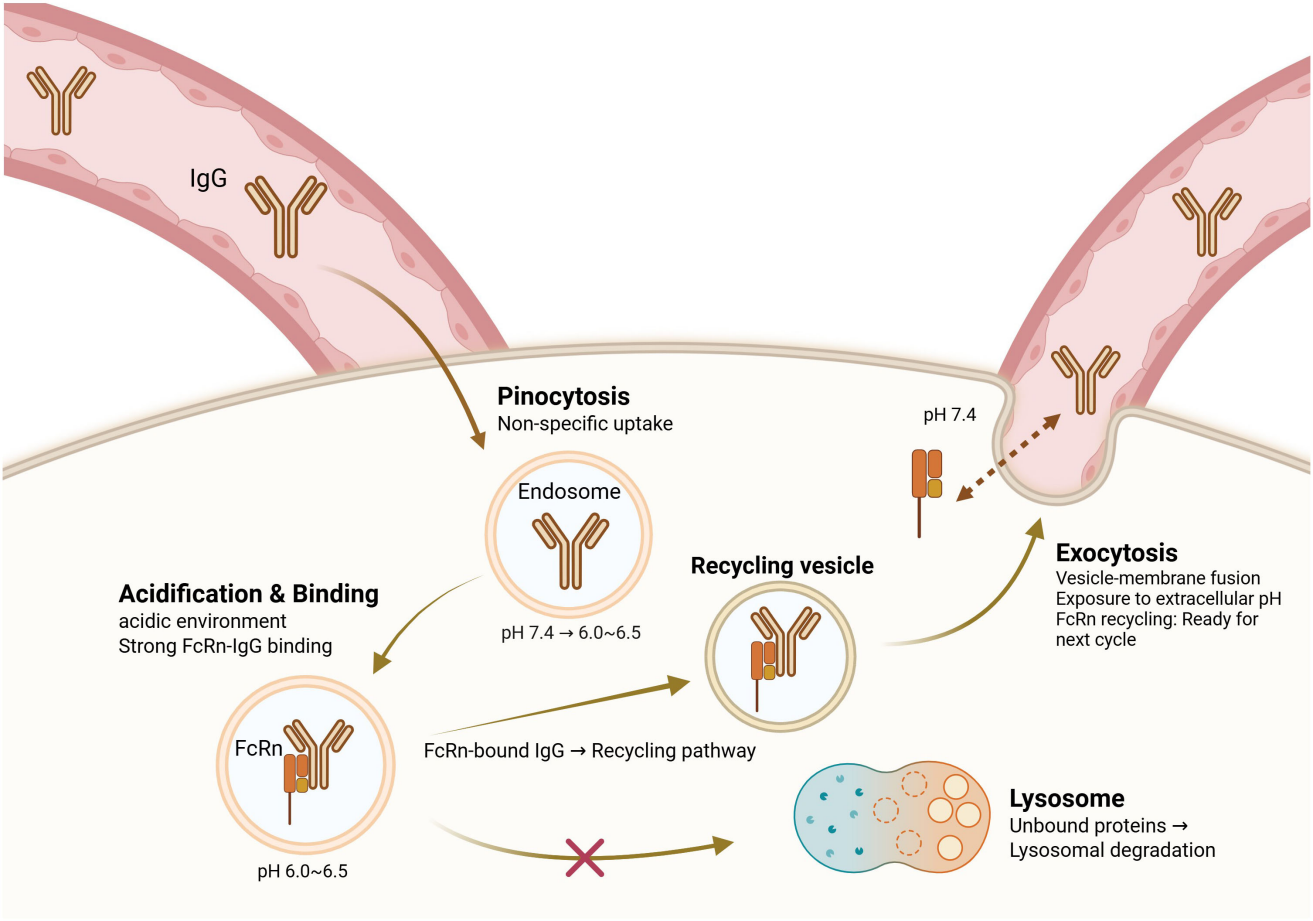
FcRn-mediated IgG recycling pathway. This figure presents a conceptual model synthesized from multiple experimental studies (5, 12, 13, 47–49). The recycling pathway depicted represents the consensus mechanism established primarily in cell culture and animal models. IgG antibodies are taken up by pinocytosis into endosomes, where the acidic pH (6.0–6.5) promotes binding to the neonatal Fc receptor (FcRn). FcRn-bound IgG is sorted into recycling vesicles, allowing it to bypass lysosomal degradation and be returned to the extracellular space via exocytosis at physiological pH (7.4). Unbound IgG is directed to lysosomes for degradation. This recycling mechanism prolongs the serum half-life of IgG and it plays a critical role in maintaining IgG homeostasis. Dysregulation of this pathway in obesity leads to pathological IgG accumulation in adipose tissue, thus contributing to inflammation and insulin resistance.

The neonatal Fc receptor (FcRn) has emerged as a central player in metabolic diseases via regulation of IgG homeostasis in fat. Unlike other immunoglobulin isotypes, IgG has an unusually long half-life owing to FcRn-mediated recycling, avoiding lysosomal degradation and sustaining serum antibody titers (12, 13). The FcRn–IgG recycling pathway was initially described by Brambell et al. in the 1960s; however, its role in metabolic tissues remained unknown until recently (14). As illustrated in **Figure 1**, this recycling mechanism involves pH-dependent binding of IgG to FcRn in acidic endosomes, followed by vesicular transport and exocytosis at physiological pH.

Recent studies have demonstrated that the same recycling mechanism is dysregulated in obesity, leading to pathological IgG accumulation only in white adipose tissue (5). In diet-induced obesity models, IgG deposition in epididymal white adipose tissue reaches quantitative levels up to 16 times greater than the circulating levels, whereas IgA and IgM fail to accumulate to such an extent (5). Notably, this IgG accumulation was observed in obese human adipose tissue, suggesting a specific receptor-mediated process rather than passive extravasation by this selective deposition (5). However, the precise mechanisms underlying selective adipose tissue accumulation in humans require further investigation, as most mechanistic data derive from rodent models.

The molecular basis of FcRn-IgG interactions has been extensively characterized in the past through structural studies. pH-dependent binding engineering studies revealed an FcRn affinity threshold that governs IgG recycling efficiency, with optimal pharmacokinetic outcomes requiring balanced affinity under both acidic (pH 6.0) and neutral (pH 7.4) pH conditions (15, 16). The structural determinants of subclass-specific FcRn binding have been mapped, showing that IgG1 and IgG4 exhibit the highest FcRn affinity, whereas IgG3 displays reduced binding due to structural differences at the CH2–CH3 interface (17, 18). Surprisingly, complementarity-determining regions in the Fab domain significantly affect the FcRn binding affinity, with up to 2.5-fold differences observed between antibodies of the same subclass, thus indicating that variable domains can modulate the IgG–FcRn interactions (19, 20).

The temporal dynamics of FcRn expression in obesity reveal a complex regulatory program. During obesity development, FcRn is upregulated by adipose progenitor cells to initiate local IgG deposition (5). As persistent obesity ensues, macrophage infiltration in the adipose tissue serves as the principal source of FcRn, creating an auto-sustaining pathway for IgG sequestration and inflammatory cell recruitment. Infiltrating macrophages not only express high levels of FcRn, but also secrete inflammatory mediators that recruit more immune cells, and the accumulated IgG enhances macrophage activation by binding to Fc receptors, thereby augmenting both IgG retention and inflammation as a positive feedback mechanism. This temporal reversal of FcRn cell sources is a transition from metabolic tissue dysfunction to a full-blown inflammatory pathology.

The functional significance of IgG deposition in adipose tissue extends beyond simple inflammatory cell recruitment. Using high-level artificial intelligence-aided molecular modeling supported by experimental validation, researchers have determined the physical interactions between the Fc CH3 domain of IgG and the insulin receptor ectodomain (5). This new mechanism of insulin resistance does not require the phosphorylation of traditional inflammatory mediators or insulin receptor substrates.

The selectivity of this molecular interaction is remarkable because bound IgG sterically hinders insulin from binding to its receptor without inducing other receptor tyrosine kinases (5). This selectivity may explain why FcRn-mediated IgG accumulation produces dramatic metabolic consequences without substantially disturbing other cellular processes. The reversibility of this process, which has been demonstrated by FcRn antagonist studies using antisense oligonucleotides, suggests that this pathway can be manipulated for therapeutic purposes, even once metabolic derangement is established (5).

The demonstration of direct IgG-insulin receptor interactions complements the prevailing model of insulin resistance caused by inflammatory cytokine interference with insulin signaling cascades. These mechanisms are not mutually exclusive, but may coexist and converge on the same pathological outcome. Instead, it revealed a mechanism whereby antibodies directly compete with hormones for receptor binding, which is a novel form of molecular mimicry in metabolic diseases. This finding may explain why conventional anti-inflammatory treatment approaches are only partially effective in treating insulin resistance, as they fail to address this antibody-mediated component of the pathophysiology. It also raises the question of whether IgG accumulation in adipose tissue reflects only FcRn-dependent recycling or is compounded by increased local antibody production. Adipose-resident B cells expand in obesity and may contribute to the elevated IgG pool, though the relative quantitative contribution remains unresolved (21, 22).

While the direct IgG-insulin receptor competition model provides a compelling mechanism (5), alternative explanations warrant consideration. Some studies suggest that adipose tissue inflammation and cytokine signaling may be sufficient to induce insulin resistance independent of IgG accumulation (88, 89). Additionally, the relative contribution of direct receptor interference versus Fc receptor-mediated inflammatory signaling remains incompletely resolved. Further research distinguishing these mechanisms—particularly in human systems—will be essential for therapeutic targeting.

Moreover, the role of adaptive immunity in metabolic homeostasis may be more nuanced than a simple pathogenic model suggests. Studies in lean mice demonstrate that regulatory T cells accumulate in visceral adipose tissue and maintain metabolic homeostasis through anti-inflammatory mechanisms (158). Similarly, certain antibody populations, including natural IgM antibodies, may play protective roles in metabolic regulation (159). These findings suggest that complete immune suppression or antibody depletion strategies should be approached cautiously, as they may disrupt beneficial immunometabolic functions alongside pathogenic ones.

### 2.2 Aging: chronic IgG accumulation and tissue fibrosis

Although obesity is an acute paradigm of IgG-induced metabolic derangement, aging provides a chronic model that exposes additional facets of IgG pathophysiology. During healthy aging, IgG slowly accumulates in adipose tissue and may cause many of the changes traditionally attributed to aging (23). The kinetics of IgG deposition with age occur over months to years, causing persistent exposure that triggers pathological processes distinct from those arising from the acute accumulation of IgG associated with diet-induced obesity. **Table 1** summarizes the key differences between obesity-associated and aging-associated IgG pathology, highlighting distinct timelines, mechanisms, and therapeutic implications.

**Table 1:** Comparison of IgG pathology in obesity versus aging.

Feature	Obesity	Aging
Timeline	Weeks (acute)	Years (chronic)
Primary outcome	Insulin resistance via insulin receptor interference (5)	Fibrosis via transforming growth factor-β and collagen deposition (19, 20)
FcRn cellular source	Early: adipose progenitors; Late: infiltrating macrophages (5)	Tissue-resident macrophages (19)
Mechanism	Direct competition of IgG with insulin for receptor binding (5)	Chronic FcR signaling leads to fibrotic remodeling (19)
Therapeutic implications	FcRn antagonism reverses insulin resistance (5)	FcRn ablation or antagonism extends healthspan/lifespan (19)
Inflammation role	Amplifies acute inflammation (5)	Drives chronic fibrotic response (19)
Reversibility	Yes—rapid improvement with FcRn antagonism (5)	Yes—but slower; requires sustained intervention (19)

This comparison synthesizes distinct experimental findings from obesity (5) and aging (23) models. While these features are documented independently, their direct temporal relationship within the same experimental system has not been comprehensively evaluated.

The single most important role of persistent IgG exposure in aging adipose tissue is to trigger progressive fibrosis, a pathological characteristic that differentiates age-associated from obesity-associated metabolic dysfunction (23). IgG engages tissue-resident macrophages via Fc receptor signaling cascades to produce high levels of transforming growth factor-β, resulting in collagen deposition (23, 24). This fibrotic pathway diverges from the acute inflammatory processes usually linked to obesity, forming a chronic remodeling program that reorganizes the core architecture of the tissue.

The fibrotic consequences of chronic IgG exposure are not merely correlated with aging but are causally associated with metabolic decline. However, whether these antibodies recognize specific antigens within adipose tissue remains poorly understood. Unlike atherosclerosis, where both protective (e.g., anti-oxLDL) and pathogenic antibodies have been described, the precise antigenic drivers of adipose-tissue IgG remain elusive (25). Treatments that reduce IgG deposition reverse metabolic dysfunction with aging (23). These findings suggest that IgG deposition is a common pathway through which several stresses of aging ultimately contribute to metabolic decline.

Caloric restriction, the most effective intervention for enhancing the healthspan and lifespan of many species, significantly reduces adipose tissue IgG deposition (23). IgG replenishment in calorie-restricted animals reverses many metabolic benefits of dietary restriction, providing unambiguous evidence for the causal role of IgG in age-related metabolic disorders. These findings suggest that the metabolic benefits of caloric restriction are partially mediated by reduced IgG synthesis or enhanced clearance mechanisms.

The therapeutic implications of these data are considerable. Conditional ablation of FcRn in macrophages alone inhibits age-related IgG accumulation and extends healthspan and lifespan of model systems (23). This myeloid-restricted approach preserves other FcRn functions, while selectively interfering with disease-induced accumulation. In addition, pharmacological modulation of FcRn by antisense oligonucleotides can restore adipose tissue function and metabolic health in old animals and provide a proof-of-concept for the therapeutic value of other interventions modulating the IgG–FcRn axis in established metabolic diseases with aging (23).

The broader implications of IgG-mediated aging of adipose tissue are potentially applicable to other age-related diseases. Since FcRn is found in many tissues, and IgG deposition could be a unifying feature of aging, the same mechanisms could be involved in cardiovascular diseases, neurodegenerative diseases, and other age-related diseases (26, 27). This suggests that targeting the IgG–FcRn pathway could have far-reaching effects beyond metabolic disease.

Obesity and aging serve as acute and chronic model of IgG accumulation, and therefore, fibrosis and insulin resistance are not mutually exclusive mechanisms. The finding that targeting FcRn not only reduces fibrotic markers but also improves metabolic dysfunction (GTT/ITT) indicators suggests relevance of aging and metabolism (23). This indicates that while obesity and aging may have distinct primary pathological mechanisms, they ultimately represent a common pathological pathway where these two mechanisms interact to cause complex metabolic dysfunction (5, 23).

Having established the fundamental role of the FcRn-IgG axis in both acute and chronic metabolic diseases, it becomes evident that IgG should not be considered as a homogeneous entity. The complexity of IgG-mediated metabolic regulation extends beyond simple quantity dependent effects to encompass sophisticated structural and functional heterogeneity that fundamentally shapes disease outcomes.

## 3 IgG subclass heterogeneity in metabolic regulation

Although the aforementioned discussion treats IgG as an homogeneous unit, emerging evidence suggests that different IgG subclasses have distinct metabolic regulatory characteristics, including subclass-specific modes of accumulation, receptor affinity, and functional consequences in obesity and aging. The four subclasses of human IgG (IgG1, IgG2, IgG3, and IgG4) possess significantly varying structural properties, Fc receptor-binding capacities, and half-life parameters, suggesting that there may be preferential alterations in the populations of individual subclasses rather than global changes in all IgG molecules.

The subclass-specific differential binding affinities of IgG for FcRn provide a mechanistic basis for explaining subclass-specific patterns of accumulation in metabolic tissues. IgG1 and IgG4 exhibit the greatest FcRn binding and longest circulation half-lives, while IgG3 exhibits significantly reduced FcRn affinity and a consequent shorter half-life owing to structural differences at the CH2–CH3 interface (17, 28). These variations in FcRn binding directly translate into differential tissue accumulation patterns following obesity onset. Previous studies have confirmed that human IgG subclasses bind to mouse FcγRs with the relative affinities IgG3 > IgG1 > IgG4 > IgG2, and that these binding patterns were remarkably similar between human and mouse ortholog receptors (29).

The most prevalent subclass that accumulates in fat tissues in diet-induced models of obesity is IgG1, which is consistent with its exceptionally high affinity for FcRn and circulating concentration (23, 30). However, the relative tissue contents of the IgG subclasses differ fundamentally from the circulating ratios, suggesting differential FcRn-mediated recycling efficiency across subclasses within the tissue environment (23). IgG2, with reduced circulating levels, shows uneven tissue accumulation in visceral fat pads, potentially because of increased local retention processes or subclass-specific interactions with tissue-resident cells.

The extended hinge region of IgG3, which is responsible for its reduced binding to FcRn, may be advantageous in certain metabolic scenarios. Although IgG3 accumulates less effectively via Fc receptor-dependent mechanisms, structural plasticity facilitates increased complement protein and Fc receptor binding, potentially resulting in divergent inflammatory responses in metabolic tissues (29, 31). Recent studies of IgG allotypes have revealed large allotype-specific variations in IgG3, with ADCC capacity affected by residues 291, 292, and 296 in the CH2 domain, and allotypic variation in hinge length affecting effector functions (32). These findings imply that FcRn-directed therapy can have subclass-specific effects on various IgG subclasses, necessitating subclass-specific therapeutic planning.

Metabolic stress leads not only to quantitative changes in total IgG but also to qualitative changes in subclass distribution, a process referred to as class switching, which may be an adaptive process that becomes maladaptive in chronic disease (33). During the pathogenesis of obesity, metabolic stress is increasingly reoriented from the anti-inflammatory IgG4 to the pro-inflammatory IgG2 subclasses as cytokines such as IFN-γ promotes switching to pro-inflammatory IgG2a, paralleling the transition from acute adaptive processes to chronic pathological inflammation (34).

IgG1, the most abundant subclass in the circulation and tissues, has the highest affinity for the insulin receptor ectodomain, and therefore could be the primary direct mediator of insulin resistance in obesity (5). Binding of high-affinity IgG1 to activating Fc receptors (FcγRI and FcγRIII) enhances macrophage activation and pro-inflammatory cytokine release, creating a tissue environment that favors continued IgG1 production and metabolic derangement reinforcement (29, 35).

In contrast, IgG4 has unique structural characteristics that may confer protective metabolic functions. The Fab-arm exchange process in IgG4 produces functionally monovalent antibodies that are incapable of activating complement or effectively forming immune complexes (36). Early in the process of metabolic stress, increased production of IgG4 may be an attempt to maintain immune homeostasis without causing inflammatory activation. Chronic metabolic stress overwhelms this protective mechanism, and pro-inflammatory subclasses predominate.

The dynamics of subclass switching also provides useful clues regarding disease pathogenesis and therapeutic timing. Prophylactic treatment when IgG4 responses remain intact may be more effective than therapy after the pro-inflammatory subclasses have become fully established. Therapies that selectively promote IgG4 production and reduce IgG1/IgG2 levels can also restore metabolic homeostasis without impairing important immune processes such as phagocytosis.

Determining subclass-specific contributions to metabolic impairment has important implications for precision medicine and drug development. Current FcRn antagonists affect all IgG subclasses equally; however, future treatments may be beneficial for subclass-selective targeting. Designing drugs that preferentially decrease IgG1 and IgG2 while preserving IgG4 may maximize the therapeutic benefit with minimal immune suppression.

Subclass profiling can also serve as a narrow biomarker for monitoring metabolic risk and therapy. The IgG1/IgG4 ratio can provide an improved measure of metabolic disease compared to total IgG measurements, particularly when the disease is in the nascent stages with normal total antibody levels. Similarly, subclass-specific glycosylation monitoring can reveal disease-specific signatures that can guide personalized treatment plans (37).

The subclass-specific perspective also enables an understanding of variations in susceptibility to metabolic diseases and responses to therapy. Genetic polymorphisms affecting subclass production, FcRn binding affinity for subclasses, or patterns of FcRn expression could explain the heterogeneity in the course of metabolic disease. The identification of these subclass-specific determinants will enable better patient stratification and treatment selection in clinical practice.

**Figure 2:**
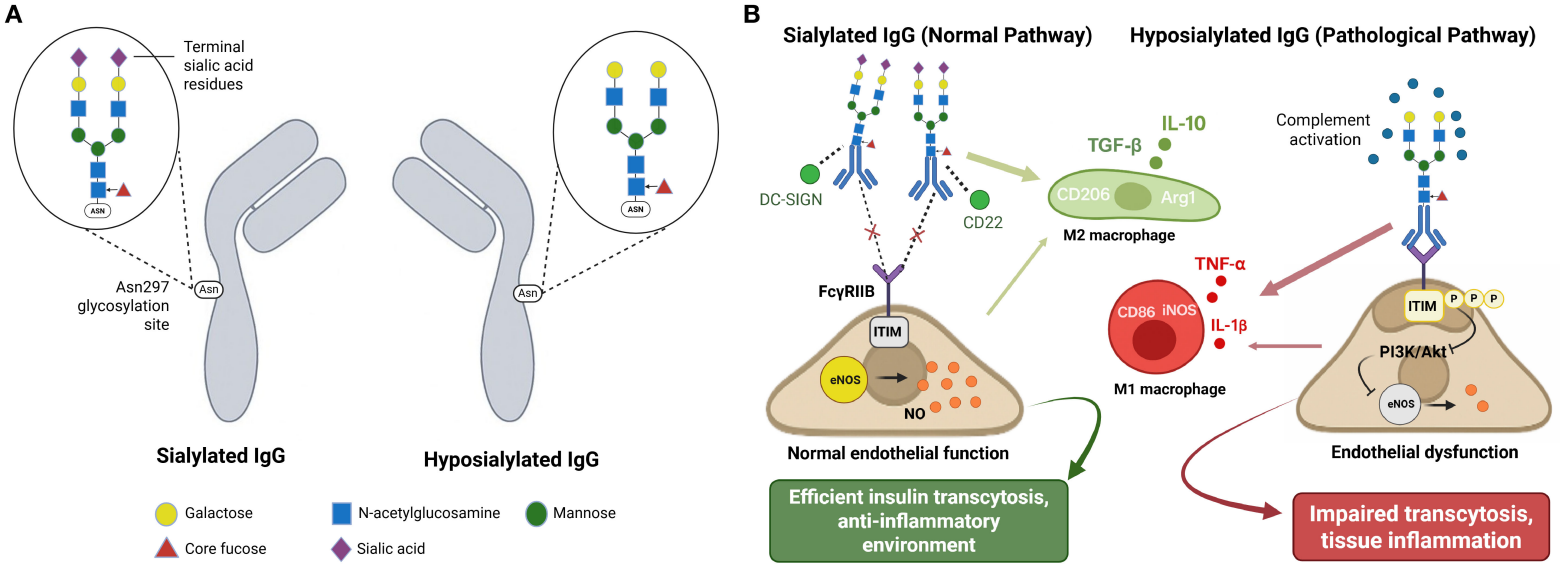
IgG glycosylation controls metabolic function through receptor-specific pathways. **(A)** IgG glycosylation structure comparison. **(B)** Glycosylation-dependent functional consequences. This schematic integrates mechanistic findings from references 6, 38, 39, 52, and 60. Panel **(A)** depicts experimentally validated glycosylation structures. Panel **(B)** represents a conceptual integration of multiple pathway studies rather than direct experimental observation of all depicted interactions simultaneously.**(A)** Structural comparison of sialylated and hyposialylated IgG. Sialylated IgG carries terminal sialic acid residues on Fc glycans at Asn297, promoting anti-inflammatory effects, whereas hyposialylated IgG lacks these residues and exhibits pro-inflammatory properties. **(B)** Sialylated IgG (normal pathway) binds to DC-SIGN and CD22, promoting M2 macrophage polarization (CD206, Arg1) with anti-inflammatory cytokine production (IL-10, TGF-β) and FcγRIIB-mediated inhibition of inflammatory signaling. This maintains normal endothelial function through preserved eNOS activity and nitric oxide production, supporting efficient insulin transcytosis. In contrast, hyposialylated IgG (pathological pathway) promotes M1 macrophage polarization (CD86, iNOS) with pro-inflammatory cytokine production (TNF-α, IL-1β), activates complement, and leads to ITIM phosphorylation and suppression of PI3K/Akt-eNOS pathway, thus resulting in endothelial dysfunction and impaired transcytosis.

IgG functional heterogeneity, beyond simple abundance, comprises post-translational modifications—chemical alterations occurring in proteins after their synthesis. More specifically, N-linked glycosylation at the conserved Asn297 position in the Fc region radically alters antibody function. Fc region glycosylation, specifically the presence or absence of terminal sialic acid residues, serves as a molecular switch to determine whether IgG exerts anti- or pro-inflammatory effects (6, 38, 39). **Figure 2A** illustrates the structural differences between sialylated and hyposialylated IgG, showing the complete versus truncated glycan chains at the Asn297 glycosylation site. Such glycosylation-dependent functional switching introduces an additional layer of complexity into IgG-mediated metabolic regulation.

The preceding discussion of subclass heterogeneity and post-translational modifications reveals that IgG populations in metabolic tissues are far from uniform. This structural and functional complexity becomes even more intricate when we consider that antibodies in adipose tissue can serve both beneficial and detrimental roles, depending on their specific properties and the physiological context in which they operate.

## 4 The dual nature of adipose tissue antibodies

### 4.1 Pathogenic versus protective IgG functions

The role of antibodies in adipose tissue biology is much more sophisticated than initially appreciated, with recent evidence suggesting that multiple populations of antibodies can be protective or pathogenic, depending on their specificity, timing, and tissue environment. This distinction discredits the simplistic models of antibody action and requires a more mature understanding of how multiple populations of antibodies affect metabolic health and diseases.

A cohort of IgG autoantibodies that bind to apoptotic adipocytes and facilitate their clearance by macrophages has recently been identified (7). Such homeostatic autoantibodies have been shown to contribute to the efficient clearance of dying or dead adipocytes, which otherwise would lead to chronic inflammation and the formation of crown-like structures (40, 41). The production of these protective autoantibodies is regulated by B cell-activating factor (BAFF), leading to dead adipocyte accumulation, increased tissue inflammation, and increased insulin resistance (7). However, the precise mechanisms underlying how BAFF specifically regulates protective versus pathogenic autoantibody production remain incompletely understood.

This protective function of autoantibodies represents a newly recognized mechanism for adipose tissue homeostasis. During physiological adipose tissue remodeling, an ongoing process with weight cycling or as a component of the normal turnover of tissues, apoptotic adipocyte clearance is required to prevent the formation of crown-like structures and the chronic inflammatory state of dysfunctional adipose tissue (42, 43). The efficacy of this clearance, or efferocytosis, is critical for maintaining tissue homeostasis and preventing the transition from acute-to-chronic inflammation (44).

The autoantibodies that facilitate this clearance are mechanistically and functionally distinct from disease-causing IgG populations that are sequestered through the FcRn pathways (45, 46). While FcRn-sequestered IgG directly affects insulin signaling, therapeutic autoantibodies facilitate the resolution of inflammation and tissue repair (5, 7). The neonatal FcRn binds to IgG in a pH-dependent manner, with high-affinity binding occurring at acidic pH and low affinity at physiological pH, enabling selective recycling of IgG (12, 47). However, FcRn does not discriminate between different IgG specificities or functional classes, and it recycles all IgG that maintain proper Fc structure and pH-dependent binding capacity (48, 49).

The protective/pathogenic status of antibody function appears to be disrupted in metabolic diseases. In obesity and old age, pathogenic IgG deposition is promoted, and the production or function of protective autoantibodies is reduced (7). This imbalance may result from multiple mechanisms, including altered B cell differentiation toward pro-inflammatory phenotypes, changes in cytokine environments that favor pathogenic autoantibody production, and impaired efferocytosis machinery that reduces the efficiency of protective autoantibody function (50, 51). However, the relative contributions of these mechanisms and their interactions remain poorly characterized, representing a critical gap for therapeutic targeting.

The differential effects of protective versus pathogenic IgG on macrophage function appear to result from both inherent IgG structural differences and concurrent changes in macrophage phenotype and receptor expression (39, 52). The metabolic environment shapes macrophage polarization, with M1-like macrophages showing enhanced responsiveness to pathogenic IgG signals, while M2-like macrophages are more responsive to protective autoantibody-mediated efferocytosis signals (53, 54). This represents a bidirectional interaction where IgG characteristics influence macrophage function, while macrophage phenotype determines IgG responsiveness.

### 4.2 IgG glycosylation and receptor specificity

The functional dichotomy between protective and pathogenic antibodies is further refined by post-translational modifications that act as molecular switches, determining the ultimate biological outcome of antibody-mediated processes. Among these modifications, glycosylation emerges as a critical determinant that can fundamentally alter antibody function independent of antigen specificity.

IgG sialylation is controlled by ST6Gal1 (β-galactoside α-2,6-sialyltransferase 1), which adds α2,6-linked sialic acids to Fc glycans at Asn297 (55, 56). While initial studies suggested IgG sialylation occurs exclusively during B cell pre-secretion (57), subsequent work demonstrated that secreted hepatocyte-derived ST6Gal1 can also modify circulating IgG using platelet-derived CMP-sialic acid (58, 59), indicating both cellular and extracellular sialylation pathways contribute to the final glycosylation profile.

The molecular determinants of the anti-inflammatory activity of sialylated IgG are subclass-specific. Terminal sialylation of IgG Fc glycans bestows anti-inflammatory activity specifically against IgG1 and IgG3 subclasses, but not IgG2 or IgG4, owing to the unique conserved amino acid residues at positions 234 and 327 in the CH2 domain (60). This subclass selectivity provides additional therapeutic opportunities for precise targeting of glycosylation-dependent IgG functions.

In obesity and type 2 diabetes, IgG is increasingly hyposialylated, losing terminal sialic acid residues that confer anti-inflammatory functions (6). Mass spectrometry analysis showed that while 19.2% of Fc Asn297-associated glycans in control IgG were terminally sialylated, no sialylation was detectable in IgG from mice fed a high-fat diet (6). This extreme loss of sialylation is a fundamental alteration in antibody structure that occurs solely in metabolic disease. However, murine IgG glycosylation is substantially different not only from human IgG but also between mouse strains (61). Therefore, these findings in mice should be interpreted as mechanistic insights rather than directly extrapolated to human IgG biology.

Hyposialylated IgG acquires the ability to engage FcγRIIB receptors—inhibitory Fc receptors that would otherwise suppress immune responses—on endothelial cells, leading to impaired insulin transcytosis and defective delivery of insulin to target tissues such as skeletal muscle (6). Insulin transcytosis is the process of the movement of insulin across the endothelial barrier from blood vessels into tissues, a necessary step for insulin to act on its target cells. This process represents tissue-specific insulin resistance at the insulin transport level, rather than insulin signaling per se. Activation of endothelial FcγRIIB by hyposialylated IgG selectively suppresses insulin-stimulated endothelial nitric oxide synthase (eNOS) activation—a nitric oxide-producing enzyme that regulates vascular function and facilitates insulin transport—disrupting the vascular component of insulin action (6, 62). **Figure 2B** demonstrates the contrasting functional consequences, showing how sialylated IgG promotes anti-inflammatory M2 macrophage polarization and maintains normal endothelial function, while hyposialylated IgG drives pro-inflammatory M1 responses and impairs insulin transcytosis.

A temporal relationship between IgG hyposialylation and metabolic dysfunction has been revealed in terms of disease progression. In model systems, IgG sialylation is normal during the first two weeks of high-fat diet feeding but then becomes progressively reduced as glucose intolerance develops (6). This time course is compatible with the potential that alterations in glycosylation are an effect rather than a cause of early metabolic dysfunction, but contribute to the progression and perpetuation of disease.

Notably, IgG glycosylation can be pharmacologically manipulated by supplementing with sialic acid precursors, such as N-acetyl-D-mannosamine (ManNAc) (6). This intervention restores IgG sialylation and preserves insulin sensitivity without affecting body weight, indicating that the metabolic effects of IgG can be dissociated from those of energy balance regulation. These findings highlight IgG glycosylation as a novel therapeutic target for metabolic diseases that does not rely on weight loss.

Regulation of IgG sialylation is complex and it involves multiple enzymatic pathways. The sialyltransferase family comprises 20 different enzymes with tissue-specific expression patterns and substrate specificities (56). In addition to ST6Gal1, other sialyltransferases, such as the ST3Gal family members, contribute to different linkage patterns of sialic acid addition (56). The balance between sialic acid addition by sialyltransferases and removal by neuraminidases (NEU1-4) creates a dynamic regulation of the cell surface and secreted protein sialylation (63, 64).

The mechanistic insights derived from experimental models provide a compelling framework for understanding IgG-mediated metabolic dysfunction, but their clinical relevance ultimately depends on validation in human populations. Fortunately, mounting clinical evidence demonstrates that the IgG abnormalities observed in laboratory studies translate meaningfully to human metabolic diseases, offering both diagnostic opportunities and therapeutic targets that extend far beyond the controlled conditions of experimental systems.

## 5 Human clinical evidence supporting IgG-mediated metabolic dysfunction

### 5.1 IgG glycosylation profiles in human insulin resistance, and diabetes

The fact that IgG glycosylation profiles can be used as biomarkers of metabolic dysfunction has important clinical utility (65, 66). IgG *N*-glycosylation profiles correlate with the degree of insulin resistance and can discriminate between insulin-sensitive and -resistant individuals, even if they are within the same BMI range (65). This suggests that glycosylation profiling of antibodies can be a more sensitive indicator of metabolic risk than traditional markers. Previous studies on autoimmune diseases have shown that sialylated IgG can modulate pathogenic immune complexes and protect against tissue injury, suggesting its potential therapeutic application in metabolic diseases associated with autoimmune components (67).

Comprehensive clinical studies have validated the association between IgG *N*-glycosylation patterns and metabolic dysfunction in human populations. A study of 313 Chinese participants stratified by homeostatic model assessment for insulin resistance (HOMA-IR) demonstrated distinct IgG *N*-glycosylation profiles across insulin-sensitive (HOMA-IR < 2.69), mildly insulin-resistant (HOMA-IR ≥ 2.69 without diabetes), and severely insulin-resistant groups (HOMA-IR ≥ 2.69 with type 2 diabetes) (68). The study revealed that IgG *N*-glycosylation modulated immune responses and reflected metabolic disorders, with canonical correlation analysis showing significant relationships between IgG *N*-glycosylation and insulin resistance–related inflammatory markers, including tumor necrosis factor-α, interleukin-6, C-reactive protein, and adiponectin (68). However, the study revealed the association between IgG N-glycosylation and insulin resistance, but causality has not yet been proven. Moreover, some population studies have reported inconsistent glycosylation patterns across different ethnic groups and age ranges (145), suggesting that additional confounding factors—including genetic background, dietary patterns, and microbiome composition—may modulate the IgG-metabolism relationship. These inconsistencies highlight the need for mechanistic validation studies.

Large-scale prospective cohort studies have established IgG glycans as predictive biomarkers of cardiometabolic diseases. The European Prospective Investigation into Cancer and Nutrition (EPIC)-Potsdam study examined 2,127 participants from a type 2 diabetes subcohort with 741 incident cases and identified specific IgG *N*-glycans associated with incident type 2 diabetes, beyond classic risk factors (69). A score based on these IgG glycan peaks showed significant association with diabetes risk across multiple validation studies (843 total cases and 3,149 total controls), with a pooled estimate of 1.50 per standard deviation increase (95% CI 1.37–1.64) (69). Moreover, consistent changes in IgG N-glycans patterns have been observed in studies on type 2 diabetes mellitus (70). These findings suggest that IgG glycosylation can play an important role as a reliable biomarker, reflecting the immunological changes that occur during insulin resistance and diabetes.

Clinical evidence also links IgG glycosylation with pregnancy-related metabolic dysfunctions. In a study of 48 pregnant women with normal glucose tolerance and 41 with gestational diabetes mellitus at 24–28 weeks of gestation, fasting insulin levels were significantly associated with several IgG glycan traits (71). These findings suggest that bisecting GlcNAc may confer functions relevant to the maintenance of glucose homeostasis, although the exact mechanisms remain to be elucidated (71).

### 5.2 Cardiovascular implications of IgG glycosylation

Clinical translation of IgG glycan biomarkers extends to cardiovascular outcomes. The JUPITER and TNT trials (comprising 162 and 397 case-control pairs, respectively) demonstrated that baseline IgG N-glycans, particularly galactosylated and sialylated forms, were associated with incident cardiovascular disease, with protective effects potentially mediated through the regulation of pro- or anti-inflammatory IgG responses (72).

IgG N-glycosylation, marked by a loss of galactose and sialic acid residues, along with increased bisecting GlcNAc, contributes to the pro-inflammatory states that drives dyslipidemia, hypertension and atherosclerosis progression (70, 73). Large cohort studies have also demonstrated that IgG glycan signatures are associated differently with the incidence of cardiovascular events based on sex (70). Galactosylated and sialylated IgG glycans showed a consistent positive correlation with HDL cholesterol and correlated inversely with LDL, triglycerides, and cardiovascular risk (72, 73). These findings highlight IgG glycan profiles as promising biomarkers for cardiovascular disease prediction and stratification.

### 5.3 Subclass-specific associations with metabolic health, and IgG levels in obesity

Human studies have also identified subclass-specific IgG glycosylation patterns associated with metabolic disorders. In a cohort of 1,826 individuals, analysis of plasma Fc glycosylation profiles of IgG1, IgG2, and IgG4 using liquid chromatography–mass spectrometry (LC–MS) demonstrated that for all subclasses, low galactosylation and sialylation and high core fucosylation were associated with poor metabolic health, including increased inflammation (C-reactive protein), reduced high-density lipoprotein (HDL) cholesterol, and elevated triglycerides (74). Notably, these associations were consistent across all three major IgG subclasses, suggesting a uniform glycosylation-dependent mechanism of metabolic regulation (74).

There are alterations in both total IgG levels and adipose tissue immune cell composition in human obesity. Clinical evidence demonstrates that obese individuals exhibit elevated IgG concentrations compared to controls, with positive correlation observed between BMI and circulating IgG levels (21, 75). Histological analysis of visceral adipose tissue from bariatric surgery patients demonstrated a 2–3 fold increase in CD19+ B cell populations, with these cells showing an activate phenotype and enhanced antibody production capacity (42, 76). The elevation of IgG and B cell population appear to be associated with the chronic low-grade inflammatory state characteristic of obesity.

### 5.4 Cross-population validation studies

The clinical relevance of IgG glycosylation extends to diverse ethnic populations. Independent Chinese populations from the Beijing Health Management and Community Cohort (442 cases, 670 controls) validated the association between IgG glycosylation profiles and type 2 diabetes, with the glycan score showing a strong association (combined odds ratio: 3.78) and improved model performance, increasing the area under the curve (AUC) from 0.74 to 0.90 (77). Studies in Chinese Muslim ethnic minorities (Kyrgyz, Tajik, and Uygur) and Han Chinese have demonstrated altered IgG N-glycan profiles in hypertension-diabetes comorbidities, suggesting the involvement of inflammatory processes and potential biomarkers for disease monitoring (78).

### 5.5 Clinical biomarker validation and clinical translation readiness

Large cross-sectional studies analyzing over 100,000 individuals have revealed associations between IgG glycome composition and numerous diseases and traits, though large interindividual variability in IgG glycome composition remains a limitation for diagnostic applications (65, 79). Despite this variability, IgG glycans demonstrate potential as biomarkers of biological age and can discriminate between insulin-sensitive and insulin-resistant individuals, even among those with similar body mass index (BMI) ranges (65, 68).

IgG glycosylation analysis represents a promising complement to existing disease biomarkers, with the knowledge of the IgG glycosylation potentially improving vaccination and immunotherapy protocols (65, 79). Its clinical utility is supported by evidence that IgG glycans serve as excellent biomarkers of biological age, with glycosylation patterns influenced by both genes and the environment, making them indicators of a person’s general health state (66, 71, 80).

## 6 Integrated damage-response networks

### 6.1 Adipocyte death and inflammatory cascades

The recognition of adipocyte death as a central trigger of metabolic inflammation has provided an important context for understanding how IgG-mediated mechanisms are integrated with other pathological processes in obesity and aging. Adipocyte death through apoptosis or necrosis releases various danger-associated molecular patterns (DAMPs) that activate innate immune responses and perpetuate tissue inflammation (81, 82).

Cell-free DNA released from dying adipocytes is one of the most important DAMPs in metabolic diseases (83, 84). This extracellular DNA acts as an endogenous ligand for Toll-like receptor 9 (TLR9), leading to macrophage activation, cytokine production, and enhanced recruitment of inflammatory cells to the adipose tissue. Direct evidence has demonstrated the accumulation of single-stranded DNA in macrophages within the obese adipose tissue, providing mechanistic insights into how adipocyte death perpetuates chronic inflammation (83).

TLR9 plays a complex role in regulating adipose tissue inflammation and metabolic homeostasis. Paradoxically, TLR9-deficient mice show increased weight gain, severe glucose intolerance, and enhanced adipose tissue inflammation when fed a high-fat diet, indicating that TLR9 signaling may have protective functions in regard to metabolic regulation (85). However, obesity-induced DNA release from adipocytes stimulates chronic inflammation and insulin resistance through TLR9 activation, suggesting context-dependent effects of this pathway (83).

The TLR9-mediated response to cell-free DNA creates an inflammatory environment that favors increased IgG production by infiltrating B cells and enhanced FcRn expression by tissue-resident cells. This creates a feed-forward loop that amplifies both DNA- and IgG-mediated inflammatory responses (21, 83). The temporal sequence of these events suggests a hypothesis that DNA release provides the initial inflammatory stimulus, whereas IgG accumulation sustains and amplifies the response over time.

In aging, this integration becomes even more complex, as age-related increases in cellular senescence and mitochondrial dysfunction compound both DAMP release and antibody production. Senescent adipocytes in aged tissues show increased susceptibility to death and enhanced DAMP release, while age-related changes in B cell function and antibody repertoire may predispose to pathological IgG accumulation. This dual burden helps explain why metabolic dysfunction accelerates with age and why interventions targeting either pathway alone may be insufficient in elderly populations (86, 87).

Experimental evidence supports this integrated model of DAMP- and antibody-mediated inflammation. TLR9-deficient mice show reduced adipose tissue inflammation and improved insulin sensitivity in response to a high-fat diet, as well as reduced macrophage infiltration and altered adipose tissue remodeling (83). Similarly, interventions that reduce IgG accumulation can break the cycle of chronic inflammation even when the initial DAMP-mediated triggers persist (5).

The interaction between DNA–TLR9 signaling and the IgG–FcRn pathway represents a convergence of innate and adaptive immune mechanisms in metabolic diseases. While TLR9 activation provides rapid inflammatory responses to tissue damage, IgG accumulation creates sustained inflammatory signaling that persists long after the initial damage occurs (5, 83). This temporal complementarity may explain why acute inflammatory responses in adipose tissue can progress to chronic metabolic dysfunction, even when the initial triggering factors are removed. Understanding this integration provides new insights into why traditional anti-inflammatory approaches, which primarily target acute mediators, have shown limited efficacy in treating chronic metabolic diseases. The persistence of antibody-mediated inflammation may explain why cytokine blockade or macrophage depletion provides only transient benefits in obesity and diabetes (88, 89).

The specificity of these responses varies significantly among the different adipose tissue depots. Visceral adipose tissue, which is more strongly associated with metabolic dysfunction than with subcutaneous fat, shows higher levels of cell-free DNA and IgG accumulation in obesity (5, 83). This depot-specific pattern may reflect differences in adipocyte susceptibility to death, local immune cell populations, or the tissue-specific expression of receptors involved in DAMP recognition and IgG binding. This depot-specific pattern aligns with well-established differences in immune cell populations between visceral and subcutaneous fat, where visceral depots show greater macrophage infiltration, increased T cell activation, and more pronounced cytokine production. The integration of IgG pathology with these known depot-specific immune differences helps explain the stronger association between visceral adiposity and metabolic risk in both obesity and aging (90, 91).

DAMPs, other than cell-free DNA, contribute to the inflammatory milieu in which IgG accumulates. Mitochondrial DNA, which contains unmethylated cytosine-phosphate-guanosine (CpG) dinucleotide motifs and short DNA sequences recognized as foreign by the immune system, similar to bacterial DNA, can also activate TLR9 and contribute to sterile inflammation in adipose tissue (92, 93). The age-related decline in mitochondrial function and increased mitochondrial DNA damage creates an additional source of DAMPs that may explain why the IgG-mediated pathology becomes more prominent with aging (94, 95).Previous studies have shown that mitochondrial DNA can interact with high mobility group box 1 (HMGB1) protein to form complexes that activate TLR9 signaling pathways, promoting cellular proliferation and inflammatory responses (84, 96). HMGB1 released from dying cells activates multiple pattern recognition receptors and enhances the inflammatory response to other DAMPs (97, 98). HMGB1 expression is significantly elevated in the adipose tissue of obese individuals compared with that in lean controls and correlates with markers of adipose tissue inflammation (98). Interactions between various DAMP signaling pathways and antibody-mediated pathways represent an area of active investigation that may reveal additional therapeutic targets.

### 6.2 Temporal dynamics of immunometabolic dysfunction

**Figure 3:**
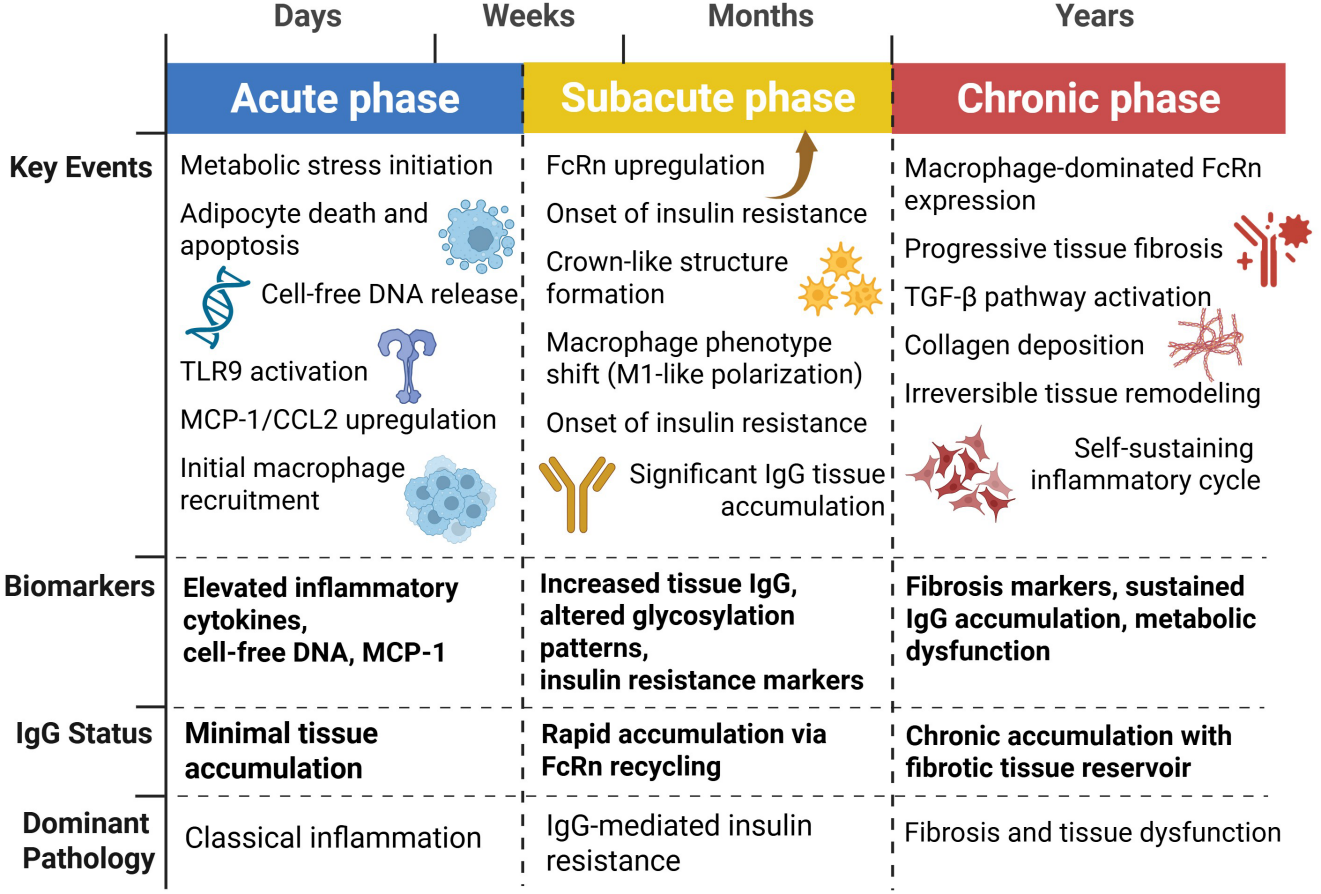
Temporal dynamics of IgG-mediated metabolic dysfunction. This temporal framework represents a conceptual synthesis of data from obesity studies (5, 40, 42, 83, 99–101) and aging studies (23, 86, 87, 102, 103). Direct longitudinal studies tracking individuals through all three phases are lacking; the timeline is extrapolated from cross-sectional and intervention studies. Metabolic inflammation follows a three-phase progression: acute phase (days-weeks) characterized by adipocyte death, DNA release, and TLR9-mediated inflammation with minimal IgG accumulation; subacute phase (weeks-months) featuring FcRn upregulation, rapid IgG tissue deposition, insulin resistance onset, and macrophage M1 polarization; and chronic phase (months-years) marked by progressive fibrosis, sustained IgG accumulation in fibrotic tissue, and self-perpetuating inflammatory cycles leading to irreversible metabolic dysfunction.

Integrating IgG-mediated pathways with established immune mechanisms requires understanding how these processes unfold over time in both obesity and aging. The classical view of metabolic immune dysfunction, focused primarily on macrophage infiltration and cytokine production, gains new complexity when antibody-mediated mechanisms are incorporated into the temporal framework. Knowledge of the chronological order of these events, as they contribute to IgG-mediated metabolic derangement, is important for delineating the intervention points and predicting disease progression. Based on existing data, a three-phase framework has been proposed for immunometabolic dysfunction: acute damage signaling, subacute antibody deposition, and chronic fibrotic remodeling. **Figure 3** illustrates this temporal progression, showing the key events, biomarkers, and dominant pathology that characterize each phase of IgG-mediated metabolic dysfunction.

The acute phase, occurring days to weeks after metabolic stress, is characterized by adipocyte death, through death receptor pathways and inflammasome activation, leading to rapid macrophage recruitment and pro-inflammatory cytokine release (40, 42, 99). Classical inflammatory mediators such as cytokines and chemokines dominate the pathology of this phase. MCP-1/CCL2 levels rapidly increase and amplify macrophage recruitment via CCR2-dependent mechanisms (100, 101). Antibodies contribute minimally during the acute phase, as IgG deposition via FcRn-based recycling is slow.

In aging, the acute phase may be less pronounced due to age-related immunosenescence, but the underlying inflammatory priming persists longer due to impaired resolution pathways. This creates a protracted acute phase that more readily transitions to antibody-mediated pathology, potentially explaining why metabolic dysfunction develops more insidiously but more persistently in elderly populations (102, 103).

The subacute phase, which lasts weeks to months, is characterized by widespread IgG deposition through FcRn-mediated mechanisms and chronic inflammatory cell infiltration (5). Dual Role of Caspase 8 in Adipocyte Apoptosis and Metabolic Inflammation (83). This phase represents the critical integration point where classical immune dysfunction becomes intertwined with antibody-mediated pathology. The established knowledge of M1 macrophage polarization, Th1 T cell responses, and pro-inflammatory cytokine networks in obesity now gains additional context as these processes both drive and are amplified by IgG accumulation (104, 105). This is when insulin resistance and crown-like formations surrounding moribund adipocytes begin to emerge (76, 106). The transition from acute to subacute may represent a therapeutic window because FcRn antagonism during this period can prevent the onset of chronic metabolic derangement.

During the subacute phase of chronic obesity, phenotypic switching of macrophage populations from a mixed acute inflammatory population to more polarized M1-like macrophages has also been observed (107, 108). Phenotypic switching is coupled with enhanced metabolic activation in macrophages, characterized by upregulation of both glycolytic and oxidative phosphorylation pathways to support their increased energy demands for phagocytosis and inflammatory responses (109, 110). IgG deposition in adipose tissue can directly cause macrophage activation and polarization via Fc receptor ligation and downstream signaling events (5, 23, 111).

The aging context adds additional complexity to the subacute phase, as age-related changes in T regulatory cell function and B cell repertoire may impair the normal resolution of inflammatory responses while simultaneously promoting pathological antibody production (112–115). This creates a perfect storm where both classical immune dysfunction and antibody-mediated pathology are enhanced and prolonged (116, 117).

The chronic phase, which persists for months to years, is characterized by progressive tissue fibrosis, impaired adipose tissue remodeling, and permanent metabolic derangement (23). During this phase, the IgG pathology becomes autogenic because the fibrotic tissue itself acts as a reservoir for the deposition of IgG and interferes with normal tissue repair processes. Extracellular matrix remodeling during this phase creates a pro-inflammatory and insulin-resistant microenvironment (118, 119).

In the context of aging, the chronic phase may represent an accelerated version of normal age-related tissue deterioration, where the combination of cellular senescence, impaired tissue repair, and antibody-mediated inflammation creates irreversible metabolic dysfunction. This integration helps explain why metabolic diseases become increasingly difficult to treat with age and why prevention strategies must target multiple pathways simultaneously (120, 121).

Interventions in the chronic stage require more invasive approaches, such as overt FcRn antagonism or antifibrotic therapy, because therapeutic processes are now self-perpetuating and established (23). The establishment of tissue fibrosis at this stage could limit the reversibility of metabolic impairment, even when effective IgG-suppressing interventions are initiated.

This temporal framework has profound implications for personalized medical approaches for metabolic diseases. Early identification of individuals shifting from the acute to subacute phase can potentially permit phase-specific interventions to avoid chronic dysfunction. Phase transition markers, including changes in IgG glycosylation or tissue IgG deposition, could be used to guide therapy duration and intensity (65, 71). Additionally, age-specific biomarkers that capture both immune cell dysfunction and antibody pathology may be necessary for optimal therapeutic monitoring in elderly populations (122).

## 7 Clinical translation and therapeutic innovation

### 7.1 FcRn modulation strategies

The hypercomplexity of IgG-mediated metabolic disruption necessitates multifaceted therapeutic approaches to counteract the different aspects of antibody biology. Clinical development efforts are currently focused on three predominant strategies: FcRn inhibition, BAFF modulation, and glycosylation reversal, each with strengths and potential limitations based on their mechanisms of action and prior clinical experience. **Table 2** summarizes these therapeutic targets for IgG-mediated metabolic dysfunction, comparing their mechanisms of action, target selectivity, immune safety profiles, and current clinical status.

**Table 2:** Therapeutic targets for IgG-mediated metabolic dysfunction: mechanisms and clinical status.

Strategy	FcRn antagonism	BAFF modulation	Glycosylation restoration	Combination therapy	Precision targeting
Representative agent	Efgartigimod (80–82)	Belimumab (90–92)	ManNAc (6, 96, 97)	Efgartigimod + ManNAc	Glycan score-guided therapy (31, 37)
Mechanism of action	Blocks FcRn-mediated IgG recycling to reduce tissue IgG (81, 82)	Inhibits BAFF to reduce B cell activation and autoantibody production (90, 91)	Supplies sialic acid precursor to restore IgG sialylation (6, 96)	Sequential FcRn blockade followed by glycan restoration	Biomarker-directed treatment selection (31, 37)
Target selectivity	IgG-specific (81, 87, 88)	Partial B cell suppression (91, 94, 95)	Functional modulation without IgG depletion (6)	Temporal IgG modulation	Patient-specific
Immune safety profile	Preserves IgA/IgM and memory B cells (81, 87, 88)	Maintains immune competence (91, 93)	Minimal off-target effects (6, 96)	Preserves beneficial antibodies while reducing pathogenic load	Individualized risk-benefit
Clinical status	Approved (autoimmune); Preclinical (metabolic) (80–82)	Approved (SLE); requires metabolic dose optimization (90–92)	Clinical trials (GNE myopathy); preclinical (metabolic) (96, 97)	Preclinical (concept)	Clinical validation needed (31, 37)

BAFF, B cell activating factor; FcRn, neonatal Fc receptor; GNE, UDP-N-acetylglucosamine 2-epimerase/N-acetylmannosamine kinase; Ig, immunoglobulin; ManNAc, N-acetylmannosamine; SLE, systemic lupus erythematosus

FcRn antagonism is the most direct approach for reducing pathological IgG deposition in metabolic tissues. Efgartigimod, an FcRn antagonist initially developed to treat autoimmune conditions such as myasthenia gravis and immune thrombocytopenic purpura, has shown promising efficacy in preclinical models of metabolic diseases (5, 23, 123). By blocking IgG recycling, efgartigimod lowers tissue IgG levels and increases insulin sensitivity without significantly affecting other immune reactions or immunoglobulin isotypes (48, 124).

Clinical trials have demonstrated both the safety and efficacy of efgartigimod in the treatment of multiple autoimmune diseases in which pathogenic autoantibodies drive disease (123, 125–128). However, efgartigimod has not yet been tested in metabolic disease populations, and extrapolation of efficacy from autoimmune to metabolic indications requires caution. The safety profile in metabolic patients—who may have different comorbidity patterns and longer treatment durations—remains to be established through dedicated clinical trials. In generalized myasthenia gravis, efgartigimod rapidly reduced total IgG and autoantibody levels in all patients, with 75% showing rapid disease improvement within one week of treatment (125, 126). Phase 2 studies on primary immune thrombocytopenia showed that efgartigimod induced up to 63.7% reduction in total IgG levels, which was associated with clinically relevant increases in platelet counts and reduced bleeding episodes (127, 128). This drug has been approved for the treatment of myasthenia gravis and is under investigation for multiple autoimmune conditions (48).

FcRn antagonistic selectivity for IgG over other immunoglobulin classes preserves the essential immune protection by IgA and IgM without interfering with the pathogenic mechanisms underlying metabolic diseases (129, 130). The clinical application of efgartigimod in autoimmune diseases has been characterized by an acceptable safety profile, with the sole significant concern being the increased infection risk due to reduced circulating levels of IgG (127, 131). However, the reversible nature of IgG suppression and perpetuation of memory B cell responses suggests that the infection risk could be acceptable in metabolic disease applications.

Extensive clinical safety data from 715 participants across multiple trials, representing over 850 patient-years of follow-up, have demonstrated that efgartigimod was well-tolerated for different dosing regimens. The pivotal ADAPT phase 3 trial demonstrated comparable treatment emergent adverse event rates between efgartigimod and placebo (77.4% vs 84.3%) with consistently low discontinuation rates (3.6% in both groups) (123). Critical for metabolic applications, efgartigimod treatment did not reduce albumin levels despite the role of FcRn in albumin recycling and did not increase LDL cholesterol levels, supporting a therapeutic window for metabolic disease applications (123, 132).

### 7.2 BAFF modulation strategies

BAFF modulation provides a more subtle strategy designed to maximize rather than eliminate antibody responses. As both the protective and pathogenic functions of antibodies in adipose tissue biology are likely lost with complete B cell depletion (7), it is possible that the titrated antagonism of BAFF might minimize pathogenic autoantibody formation while maintaining protective autoantibodies that support adipocyte clearance and tissue homeostasis.

Belimumab, a licensed BAFF antagonist for systemic lupus erythematosus, has established a clinical precedent for targeted BAFF modulation (133–135). In patients with lupus, belimumab blocks autoantibody production and B cell activation while maintaining overall immune competence, demonstrating that selective modulation of BAFF can provide therapeutic benefit while retaining key immune functions (134, 136). The mechanism involves neutralizing soluble BAFF and preventing its interaction with BAFF receptors, leading to the selective depletion of naïve and activated B cells while preserving memory B cells (137, 138). However, the optimal dosing regimen for metabolic disease applications may differ substantially from that used in autoimmune conditions, as the goal is partial, not complete, BAFF neutralization (7). This would require careful dose optimization to balance the protective and pathogenic activities of antibodies, and potentially individualized dosing based on patient-specific antibody profiles.

### 7.3 Glycosylation modulation strategies

Glycosylation correction induced by sialic acid precursor supplementation is a promising strategy to restore IgG function without altering antibody levels (6). ManNAc supplementation has been effective in restoring IgG sialylation and improving insulin sensitivity in preclinical models, without affecting body weight or overall antibody production (6). This approach is extremely desirable because it corrects the functional properties of circulating antibodies without modifying their synthesis or clearance.

The clinical development of ManNAc in its metabolic indications is based on previous experience with sialic acid supplementation for hereditary inclusion body myopathy, in which ManNAc was shown to be clinically effective and safe (139, 140). In this orphan genetic disease, in which sialic acid biosynthesis is defective, supplementation with ManNAc restores cellular sialylation and improves muscle function, establishing both the safety profile and the concept that sialic acid precursors can cure glycosylation defects in human disease. The metabolic activities of ManNAc are specifically mediated by IgG resialylation and not by the general effects on cellular sialylation, suggesting a direct mechanism with fewer side effects (6).

Alternative approaches for modulating IgG sialylation include enzymatic modifications using recombinant sialyltransferases or neuraminidases. *In vitro* glycoengineering methods using β-1,4 galactosyltransferase-1 and ST6Gal1 have been successfully applied to produce homogeneously sialylated antibodies (141). Mammalian cell line glycoengineering through overexpression of ST6GAL1 and manipulation of the CMP-sialic acid transporter can generate highly sialylated IgG glycoforms (142).

Combination treatment approaches may prove to be the most appropriate for the multidimensional nature of IgG-mediated metabolic dysregulation. Targeting multiple aspects of antibody biology simultaneously or sequentially may result in synergistic activity, with less risk from any single intervention (143). For example, an initial FcRn antagonist administration to reduce pathological IgG accumulation could be followed by supplementation with ManNAc to improve the functional attributes of the newly produced antibodies.

## 8 Precision medicine approaches

**Table 3** summarizes key biomarkers for precision medicine approaches in IgG-mediated metabolic dysfunction, including their methods, clinical roles, and performance data. The metabolic dysfunction heterogeneity induced by IgG suggests that precision medicine approaches may be essential for achieving maximum therapeutic efficacy. IgG glycosylation analysis represents a promising biomarker for patient stratification, with demonstrated ability to discriminate between insulin-sensitive and insulin-resistant individuals beyond traditional risk factors such as BMI and age in research cohorts (65, 68). However, clinical validation for therapeutic decision-making is lacking. No prospective trials have demonstrated that glycosylation-guided treatment selection improves outcomes compared to standard approaches, and the substantial interindividual variability in IgG glycome composition (65, 79) currently limits diagnostic application without standardized reference ranges. Clinical validation in the Beijing health management cohort showed remarkable discrimination performance, with AUC improving from 0.74 (clinical traits alone) to 0.90 when glycan profiling was added (77).

**Table 3:** Key biomarkers for precision medicine in IgG-mediated metabolic dysfunction.

Biomarker	IgG glycosylation profiling	IgG glycan score	Subclass-specific IgG glycosylation	Cross-ethnic glycan patterns	Cardiovascular glycan signatures	Gestational diabetes glycans	Tissue IgG imaging	FcRn polymorphisms
Method	Mass spectrometry, microarray (37, 38)	LC–MS composite scoring (32, 34)	LC–MS subclass profiling (33)	Standardized LC–MS (35)	JUPITER/TNT protocols (36)	Plasma protein glycan analysis (39)	PET tracer, biopsy (105)	SNP genotyping (15, 106, 107)
Clinical role	T2DM risk stratification; insulin resistance classification (31, 37)	Diabetes prediction; early intervention targeting (32, 34)	Metabolic phenotyping; inflammation assessment (33)	Global applicability; population-specific risk (35)	CVD risk prediction in metabolic disease (36)	Pregnancy metabolic risk assessment (39)	Localized disease activity; FcRn-targeted therapy guidance (105)	Therapy response prediction; dosing optimization (15, 106, 107)
Pros	Non-invasive, predictive, blood-based (37, 38)	Strong association (OR 3.78); multi-population validation (32, 34)	Consistent across IgG1/IgG2/IgG4; HDL correlation (33)	Validated across Asian populations; ethnic minorities (35)	Galactosylated/sialylated IgG protective; HDL correlation (36)	Early detection; glucose homeostasis correlation (39)	Site-specific info, response tracking, real-time (105)	Stable, predictive, blood-based, pharmacogenomic (15, 106, 107)
Cons	Needs standardization; population variations (37, 38)	Requires population-specific cutoffs (32, 34)	Complex analysis; higher cost (33)	Population-specific variations (35)	Limited to specific glycan types (36)	Limited to pregnancy; mechanism unclear (39)	Invasive or in development (105)	Limited functional data; population-specific (15, 106, 107)
Performance data	AUC improved 0.74→0.90 with NRI: 0.35; IDI: 0.42; Validated in 1826 Chinese Han and minority populations (31, 37)	843 cases validated across 2,127 individuals prospective design (32, 34)	Consistent across IgG1/IgG2/IgG4 subclasses (33)	Validated across Kyrgyz, Tajik, and Uygur populations (35)	162–397 case-control pairs; galactosylated/sialylated forms protective (36)	48 controls vs 41 GDM cases; glucose homeostasis correlation (39)	Up to 16-fold adipose accumulation vs plasma (5, 105)	715 participants, >850 patient-years safety data (80, 101, 102)

AUC, area under the curve; ADAPT, autoimmune disease antibody-mediated pathology trial; ADCC, antibody-dependent cellular cytotoxicity; CVD, cardiovascular disease; FcRn, neonatal Fc receptor; GDM, gestational diabetes mellitus; HDL, high-density lipoprotein; IDI, integrated discrimination index; IgG, immunoglobulin G; LC–MS, liquid chromatography–mass spectrometry; NRI, net reclassification index; OR, odds ratio; PET, positron emission tomography; SNP, single nucleotide polymorphism; T2DM, type 2 diabetes mellitus.

However, the clinical rationale for biomarker-guided treatment selection remains largely theoretical. While IgG glycosylation patterns can identify high-risk individuals and monitor therapeutic responses—as demonstrated by changes following weight loss interventions and anti-inflammatory therapies (37, 79, 144)—prospective trials establishing that biomarker-guided therapy improves clinical outcomes compared to standard approaches are lacking. The substantial interindividual variability in IgG glycome composition across populations also presents challenges for establishing universal reference ranges and treatment algorithms (65, 79, 145).

## 9 Future directions and emerging questions

The recognition of IgG as a crucial mediator of metabolic dysfunction creates numerous avenues for future research that fall outside the boundaries of our current understanding of antibody biology in metabolic diseases. Several significant questions remain to be answered before the full therapeutic potential of inhibiting IgG-mediated pathways can be accomplished and their broader implications for human health can be realized.

The role of other classes of immunoglobulins in metabolic regulation is only partly understood despite new evidence that they play a significant role in metabolic homeostasis via alternative mechanisms. Although IgM and IgA are not FcRn-bound and therefore do not accumulate by the same mechanism as IgG, new evidence suggests that IgA regulates intestinal metabolism and glucose homeostasis through alternative mechanisms (146, 147). IgA-deficient mice show decreased glucose tolerance and insulin sensitivity during high-fat diet feeding, suggesting an important role of this family of immunoglobulins in metabolic regulation (146). Studies have demonstrated that sialylated IgM can inhibit T cell proliferation through internalization via FcμR, whereas asialylated IgM lacks this inhibitory capacity, indicating glycosylation-dependent functional regulation extends beyond IgG (148).

The intestinal immune system is important for investigating the regulation of immunoglobulin-mediated metabolic control. Secretory IgA in the gut plays an important role in maintaining microbial homeostasis and intestinal barrier function, both of which strongly correlate with metabolic health (146, 147). The interaction of intestinal IgA responses with systemic IgG-mediated disease is a potentially important axis of therapeutic intervention, particularly in light of the new appreciation of the gut–adipose tissue axis of metabolic disease (149, 150).

Tissue-specific differences in FcR expression and function are critical for future studies. Various tissues have different patterns of Fc receptor expression, which may dictate the tissue-specific effects of antibody accumulation (145, 151). It would be valuable to understand how receptor expression patterns dictate the anatomical distribution of IgG-mediated pathology to guide therapeutic strategies that preferentially protect metabolically vital tissues without compromising immune function.

Given the increased focus on metabolic dysregulation in neurodegenerative diseases, the brain is a particularly intriguing target tissue for IgG-mediated pathology (152). FcRn expression has been described in the blood–brain barrier and could potentially permit the transport of IgG across the central nervous system. Therefore, similar accumulation mechanisms may be the basis for neurometabolic dysfunction (153). The theoretical connection between IgG-mediated metabolic pathology and cognitive impairment is an important research topic.

The potential of immunometabolic dysfunction as a causative factor in other aging diseases apart from diabetes and obesity is an interesting field to explore. As IgG deposition has been postulated to be a phenomenon of aging, it may be argued that common mechanisms are involved in cardiovascular disease, cancer, and other age-related diseases (23, 144, 154). Elucidating the common immunometabolic pathways between two or more age-related diseases may reveal the general mechanisms of aging and suggest broader applications for IgG-targeted therapeutic interventions.

Cardiovascular disease is a highly promising candidate for study given the established associations between inflammation, insulin resistance, and atherosclerosis (155, 156). The potential involvement of IgG deposition in vascular tissues and its role in endothelial dysfunction and atherosclerotic plaque formation may be novel therapeutic targets for cardiovascular disease prevention and treatment.

The simultaneous design of combination therapies targeting various facets of immunometabolic derangement is crucial for clinical progress. Given the multicomponent nature of the IgG-related pathology and its intersection with other inflammatory and metabolic pathways, optimal therapeutic responses may require interventions that simultaneously affect multiple targets. The synergistic potential of combining FcRn blockade, glycosylation alteration, and traditional metabolic therapy will be central to translating mechanistic insights into better clinical outcomes (157).

The timing and order of combination interventions may be particularly important given the temporal dynamics of immunometabolic dysfunction. Varying approaches may be required for early treatment in the acute or subacute phases versus the treatment of chronic diseases after onset (5, 83). The development of treatment algorithms using biomarkers to determine the optimal timing and combination of interventions is a key challenge for clinical applications.

Finally, the possibility of the preventive use of IgG-targeted therapies is an uncharted field with far-reaching public health consequences. Since IgG deposition antedates overt metabolic diseases, prevention of diabetes and other metabolic diseases in high-risk subjects may be possible through early intervention (20, 65). Identification of predictive biomarkers of impending IgG-driven pathology prior to the onset of clinical symptoms would allow for the development of truly preventive regimens for metabolic diseases.

### 9.1 Limitations and future directions

Several limitations of the current evidence base warrant acknowledgment. First, much of the mechanistic data linking IgG accumulation to metabolic dysfunction derives from rodent models, particularly mice. While these provide valuable insights, IgG glycosylation patterns and subclass distributions differ substantially between mice and humans (61), potentially limiting direct translatability. Second, the temporal framework proposed here synthesizes cross-sectional and intervention studies from obesity and aging separately; longitudinal studies tracking IgG dynamics through metabolic disease progression in the same individuals are lacking. Third, causality remains incompletely established in human populations, where most evidence is associative. While animal studies demonstrate causation through genetic manipulation and antibody transfer experiments (5, 7, 23), human intervention trials with IgG-targeted therapies in metabolic disease are absent.

Fourth, alternative mechanisms may explain observed associations. For instance, IgG accumulation could represent an epiphenomenon of underlying metabolic dysfunction rather than a primary driver. Classical mechanisms of insulin resistance—including intracellular lipid accumulation (particularly diacylglycerol and ceramides), mitochondrial dysfunction, and endoplasmic reticulum stress—remain well-established primary drivers of metabolic disease with extensive mechanistic validation (160). While IgG accumulation correlates with metabolic dysfunction, the temporal sequence and causal hierarchy relative to these established mechanisms require clarification. The relative contributions of direct receptor interference, Fc receptor-mediated inflammation, and other immune pathways remain to be quantified. Fifth, the precision medicine applications proposed require substantial further validation, including prospective clinical trials demonstrating that biomarker-guided therapy improves outcomes.

These limitations highlight critical research needs (1): longitudinal human studies tracking IgG glycosylation, tissue accumulation, and metabolic parameters simultaneously (2); mechanistic studies in human tissue systems to validate rodent-derived mechanisms (3); clinical trials of IgG-targeted therapies in metabolic populations (4); standardization of glycosylation analysis methods and establishment of clinically validated reference ranges; and (5) comparative studies directly evaluating IgG-mediated versus alternative mechanisms within the same experimental systems.

## 10 Conclusion

Recognition of IgG as a central mediator of metabolic dysregulation represents a paradigm shift that fundamentally alters our understanding of the relationship between immune system activity and metabolic health. The convergence of obesity, aging, and autoimmunity research has revealed that antibodies are not merely passive markers of immune activation, but are active promoters of metabolic illness via direct molecular engagement with core metabolic signaling pathways.

The chronological progression from acute inflammatory reactions to subacute IgG deposition and chronic fibrotic dysfunction provides a unified schema for the development and persistence of metabolic diseases over time. This model explains why traditional anti-inflammatory approaches have been unsuccessful in the management of established metabolic diseases, as they fail to address the antibody-mediated components that dominate later stages of the disease. The fact that different populations of antibodies can exert opposing effects on metabolic health highlights the complexity of antibody biology and the need for nuanced therapeutic approaches.

Identification of the exact molecular targets in IgG-mediated mechanisms offers realistic opportunities for therapeutic interventions based on established pharmaceutical approaches. The clinical applications of FcRn antagonists, BAFF modulators, and glycosylation-restoring therapies offer multiple complementary approaches to target different aspects of IgG-mediated metabolic dysfunction. The encouraging safety profiles of these interventions for autoimmune disease suggest that translation to metabolic indications is possible with cautious dose optimization and patient selection.

Precision medicine principles are required to optimize therapeutic results as these approaches move toward the clinic. IgG-mediated disease heterogeneity demands individualized approaches based on patient-specific factors, such as antibody glycosylation status, tissue-specific accumulation patterns, and genetic variation in the implicated receptors and enzymes. Biomarker-based treatment algorithms are required to establish the optimal timing, dosage, and combination of therapies for a particular patient.

Most importantly, the broader implications of these findings extend beyond metabolic diseases and impinge on our understanding of aging, immunity, and the fundamental mechanisms that maintain tissue homeostasis throughout life. The recognition that antibodies can flip from protective to pathogenic functions provides new insights into how beneficial immune responses can become maladaptive in chronic diseases. As we continue to clarify the complex interplay between antibodies and metabolism, we may discover that many aging-related diseases share immunometabolic mechanisms that can be treated using similar therapies.

Clinical translation of these advances represents both a challenge and an opportunity. Although the identification of specific molecular targets has therapeutic potential, the complexity of antibody biology and the need for individualized approaches necessitate novel clinical trial designs and regulatory strategies. It is also critical to recognize that many IgG-mediated mechanisms have been elucidated in murine models, in which IgG glycosylation patterns and subclass distributions differ from humans. Therefore, comprehensive investigations of IgG glycosylation in human tissues are required prior to clinical translation. The ultimate success of these efforts depends on the continued collaboration between basic scientists, clinical investigators, and pharmaceutical developers to ensure that mechanistic insights are efficiently translated into improved patient outcomes.

Looking ahead, the field of immunometabolism remains poised to advance rapidly, with emerging findings relentlessly challenging existing paradigms. The role of IgG in metabolic diseases offers a compelling example of how distinct biological systems can engage in unforeseen ways to influence human health. The continued exploration of these interactions promises to provide new insights into disease mechanisms and therapeutic opportunities that can transform our approach to metabolic diseases and aging-disorder therapies.
